# Struktur und Einrichtung des Deutschen Cochlea-Implantat-Registers (DCIR)

**DOI:** 10.1007/s00106-023-01309-7

**Published:** 2023-05-17

**Authors:** T. Stöver, S. K. Plontke, O. Guntinas-Lichius, H-J. Welkoborsky, T. Zahnert, K. W. Delank, T. Deitmer, D. Esser, A. Dietz, A. Wienke, A. Loth, S. Dazert

**Affiliations:** 1 Klinik für Hals‑, Nasen‑, Ohrenheilkunde, Universitätsklinikum Frankfurt, Theodor-Stern-Kai 7, 60590 Frankfurt, Deutschland; 2 Klinik für Hals‑, Nasen‑, Ohrenheilkunde, Kopf- und Halschirurgie, Universitätsmedizin Halle, Halle (Saale), Deutschland; 3 Klinik für Hals‑, Nasen- und Ohrenheilkunde, Universitätsklinikum Jena, Jena, Deutschland; 4 Klinik für Hals‑, Nasen‑, Ohrenheilkunde, Klinikum Nordstadt, Hannover, Deutschland; 5 Klinik für Hals‑, Nasen- und Ohrenheilkunde, Universitätsklinikum Dresden, Dresden, Deutschland; 6 Hals-Nasen-Ohren-Klinik, Klinikum Ludwigshafen, Ludwigshafen, Deutschland; 7 Klinik für Hals‑, Nasen‑, Ohrenheilkunde, Universitätsklinikum Leipzig, Leipzig, Deutschland; 8 Kanzlei WBK, Rechtsanwalt Fachanwalt Medizinrecht, Köln, Deutschland; 9 Universitätsklinik für Hals‑, Nasen-, und Ohrenheilkunde, St. Elisabeth Hospital, Bochum, Deutschland

**Keywords:** Rehabilitation, Implantierbare Neurostimulatoren, Prothesen und Implantate, Zertifizierung, Qualitätssicherung, Rehabilitation, Implantable neurostimulators, Prostheses and implants, Certification, Quality control

## Abstract

Die Versorgung von hochgradig schwerhörigen oder ertaubten Menschen mit einem Cochleaimplantat (CI) ist ein sehr erfolgreicher, aber auch komplexer und lebenslanger Versorgungsprozess, der eine hohe Struktur‑, Prozess- und Ergebnisqualität erfordert. Die Verwendung von medizinischen Registern stellt ein geeignetes Instrument dar, um versorgungsrelevante Qualitätssicherung zu betreiben und gleichzeitig wissenschaftliche Daten zu erheben. Auf Initiative des Präsidiums der Deutschen Gesellschaft für Hals-Nasen-Ohren-Heilkunde, Kopf- und Hals-Chirurgie (DGHNO-KHC) sollte daher ein deutschlandweites CI-Register (Deutsches Cochlea-Implantat-Register, DCIR) eingerichtet werden. Hierzu sollten folgende Ziele erreicht werden: 1. rechtliche und vertragliche Grundlagen des Registers; 2. Definition der Registerinhalte; 3. Erarbeitung von Auswertungsstandards (klinikspezifische und nationale Jahresberichte); 4. Entwicklung eines Logos; 5. produktiver Betrieb des Registers. Nach der Definition eines Leistungskatalogs, der die Inhalte, die Struktur und den Betrieb des DCIR festlegt, wurde ein Registerbetreiber mit audiologischer Expertise gesucht. Nach Abwägung verschiedener Angebote wurde das Register schließlich in Kooperation mit dem Anbieter INNOFORCE (Ruggell, Liechtenstein) als Registerbetreiber technisch umgesetzt. Dabei wurde auch eine Schnittstelle zur Überführung von Daten aus bereits existierenden Datenbanken realisiert sowie ein Datenschutzkonzept für den produktiven Betrieb des DCIR unter wissenschaftlicher Leitung des Präsidiums der DGHNO-KHC erarbeitet. Seit Januar 2022 können von den CI-versorgenden Einrichtungen (CIVE) pseudonymisierte Daten in das DCIR eingegeben werden. Bisher haben sich 75 Kliniken aus Deutschland zur Registerteilnahme vertraglich bereit erklärt. In den ersten 15 Monaten des Registerbetriebs wurden bereits mehr als 2500 CI von mehr als 2000 Patienten erfolgreich in das DCIR aufgenommen. Die hier präsentierte Arbeit beschreibt die Strukturierung, den Aufbau und die erfolgreiche Einrichtung des DCIR. Die Einführung des DCIR stellt eine wichtige Säule einer zukünftigen wissenschaftlich basierten Qualitätssicherung der CI-Versorgung dar. Das hier dargestellte Register kann daher beispielgebend für andere Bereiche der medizinischen Versorgung betrachtet werden und setzt damit auch international Standards.

**Zusatzmaterial online:**

Die Online-Version dieses Beitrags (10.1007/s00106-023-01309-7) enthält weiteres Material: Datenblöcke des DCIR.

## Abstract (en)

Hearing rehabilitation of patients with severe hearing loss or deafness using cochlear implants (CI) is a very successful but also complex and lifelong process that requires high quality standards for structure, process, and results. Medical registries represent an ideal tool for conducting quality control relevant to care while at the same time collecting scientific data. Therefore, at the initiative of the Executive Committee of the German Society of Oto-Rhino-Laryngology, Head and Neck Surgery (DGHNO-KHC), a Germany-wide CI registry (the German Cochlear Implant Register, DCIR) was to be established. The following goals were to be achieved: 1) legal and contractual basis for the register; 2) definition of the register contents; 3) development of evaluation standards (hospital-specific and national annual reports); 4) development of a logo; 5) practical operation of the registry. After defining a catalog of services that defines the content, structure, and operation of the DCIR, a registry operator with audiological expertise was sought. After considering various offers, the registry was technically implemented in cooperation with the provider INNOFORCE (Ruggell, Liechtenstein) as registry operator. This also included the realization of an interface for data transfer from previously existing databases and development of a data protection concept for productive operation of the DCIR under the scientific leadership of the DGHNO-KHC Executive Committee. Since January 2022, it has been possible for participating hospitals to enter pseudonymized data into the DCIR. To date, 75 hospitals in Germany have contractually agreed to participate in the registry. During the first 15 months, data from over 2500 implants in over 2000 patients were registered in the DCIR. The work presented here describes the structuring, development, and successful establishment of the DCIR. Introduction of the DCIR represents an important milestone of future scientifically based quality control in CI care. The registry presented here can therefore be considered as an example for other areas of medical care and thus also sets an international standard.

## Versorgungsrelevante Qualitätssicherung

Die Versorgung von hochgradig schwerhörigen oder ertaubten Menschen mit einem Cochlea-Implantat (CI) ist ein sehr erfolgreicher, aber auch komplexer und lebenslanger Versorgungsprozess [1]. Die medizinisch-wissenschaftlichen Grundlagen einer CI-Versorgung sind inzwischen sehr standardisiert und über eine AWMF-Leitlinie (Arbeitsgemeinschaft der Medizinisch-Wissenschaftlichen Fachgesellschaften; AWMF-Register-Nr. 017-071) [2] definiert. In einem aufwendigen Konsentierungsprozess wurde die inzwischen 3. Fassung der Leitlinie im Jahr 2020 unter Federführung der Deutschen Gesellschaft für Hals-Nasen-Ohren-Heilkunde, Kopf- und Hals-Chirurgie e. V. (DGHNO-KHC) mit den ebenfalls für die CI-Versorgung wesentlichen Fachgesellschaften, der Deutschen Gesellschaft für Phoniatrie und Pädaudiologie (DGPP) und der Deutschen Gesellschaft für Audiologie (DGA), verabschiedet. Die aktuelle Leitlinie beschreibt nicht nur den derzeit in Deutschland geltenden medizinisch-wissenschaftlichen Standard der Diagnostik und der operativen und postoperativen Therapie, sondern auch den notwendigen, lebenslangen Versorgungsprozess. Dieses Dokument ist damit nicht nur in Deutschland ein Meilenstein in der Qualitätssicherung der CI-Versorgung, sondern weltweit. Erstmals wurden hier wesentliche Aspekte der Strukturqualität, der Prozessqualität und der Ergebnisqualität für die CI-Versorgung definiert. Auf Basis dieser Leitlinie wurde durch das Präsidium der DGNHO-KHC eine praktische Handlungsempfehlung zur Umsetzung der Leitlinieninhalte erarbeitet und ebenfalls gemeinsam durch die betreffenden Fachgesellschaften (DGHNO-KHC, DGPP, DGA) konsentiert (CI-Weißbuch der DGHNO-KHC) [3].

Die Qualitätssicherung eines komplexen und lebenslangen Prozesses, wie es die CI-Versorgung ist, stellt eine große Herausforderung dar. Die Endverantwortung für den lebenslangen CI-Versorgungsprozess liegt unzweifelhaft bei der Cochlea-Implantat-versorgenden Einrichtung (CIVE). In der Regel handelt es sich hierbei um eine Hauptabteilung für Hals-Nasen-Ohren-Heilkunde, Kopf- und Halschirurgie [2]. Dies ist begründet in der medizinisch-ärztlichen Verantwortung (z. B. ärztliche Indikationsstellung, chirurgische Implantation, Koordination und Verantwortung des Gesamtprozesses). Zudem bestehen rechtliche Vorgaben („Medizinprodukte-Betreiberverordnung“ – MPBetreibV), nach denen die CIVE als der „Betreiber des Implantats“ zu betrachten ist [4].

## Bedeutung von medizinischen Registern

Die Verwendung von medizinischen Registern stellt ein wirksames Instrument dar, um in diesem Kontext versorgungsrelevante Qualitätssicherung zu betreiben und gleichzeitig wissenschaftliche Daten zu erheben, die zudem die Basis zukünftiger Leitlinienentwicklungen darstellen können. Dies gilt insbesondere, wenn diese klinischen Daten nicht nur multizentrisch, sondern landesweit erhoben werden. Für eine Reihe medizinischer Implantate oder Erkrankungen werden medizinische Register bereits seit vielen Jahren sehr erfolgreich betrieben. Zu nennen sind hier u. a. das Traumaregister [5] oder auch das Endoprothesenregister [6].

Obwohl die CI-Versorgung eine in Deutschland bereits seit Ende der 1980er-Jahre eingeführte Versorgungsform darstellt, existieren bisher nur ungenügende nationale Daten zu der Zahl der insgesamt versorgten Patienten, den Komplikationen, einer herstellerunabhängigen Erfassung der Implantatsicherheit, der Langzeitstabilität einer Hörverbesserung durch das CI oder den Langzeiteffekten auf die Lebensqualität. Das Implantateregistergesetz (IRegG) [7] sieht zukünftig eine verpflichtende Dokumentation von Cochlea-Implantaten vor. Die praktische Umsetzung dieses Gesetzes für Cochlea-Implantate ist allerdings gegenwärtig im Hinblick auf einen konkreten Zeitpunkt nicht exakt absehbar.

Sowohl in der Erarbeitung der CI-Leitlinie als auch des CI-Weißbuchs wurde daher deutlich, dass die jeweils erarbeiteten Qualitätskriterien nur den aktuell bekannten wissenschaftlichen Stand repräsentieren können. Hieraus resultierte die Erkenntnis, dass nur durch die Einrichtung eines nationalen Registers langfristig wissenschaftlich relevante Fragestellungen, aber auch die Grundlagen zukünftiger Qualitätsparameter zu erarbeiten wären. Insofern stellt die Erarbeitung eines deutschlandweiten CI-Registers eine konsequente Fortsetzung der in die Zukunft gerichteten kontinuierlichen Weiterentwicklung der CI-Leitlinie und des CI-Weißbuchs und damit der weiteren Qualitätssicherung der CI-Versorgung dar.

Die inhaltlichen Grundzüge des hier dargestellten CI-Registers wurden bereits für die 2. Fassung des CI-Weißbuchs im Mai 2021 durch die DGHNO-KHC erarbeitet [3]. Auf Initiative des Präsidiums der DGHNO-KHC sollte daher auf der Grundlage der AWMF-CI-Leitlinie und des CI-Weißbuchs ein deutschlandweites CI-Register (Deutsches Cochlea-Implantat-Register, DCIR) aufgebaut werden. Hierzu sollten folgende Ziele erreicht werden:

1. Erarbeitung der rechtlichen und vertraglichen Grundlagen zur Einrichtung und zum Betrieb eines klinischen Registers unter wissenschaftlicher Leitung der DGHNO-KHC
2. Definition der Registerinhalte auf der Basis der aktuellen CI-Leitlinie und des CI-Weißbuchs
3. Erarbeitung eines Auswertungsstandards (klinikspezifische und nationale Jahresberichte)
4. Entwicklung eines DCIR-Logos
5. Beginn der Dateneingabe und produktiver Betrieb des DCIR

## Material und Methoden

### Wissenschaftliche Grundlagen des DCIR

Der Prozess der Hörrehabilitation mit einem CI ist in Deutschland durch die CI-Leitlinie [2] im Detail beschrieben und berücksichtigt sowohl die Strukturqualität, die Prozessqualität und die Ergebnisqualität des kompletten Versorgungsprozesses. Diese Leitlinie wurde im Konsens der für die CI-Behandlung relevanten medizinisch-wissenschaftlichen Fachgesellschaften, der DGHNO-KHC, der DGPP und der DGA erarbeitet. Diese Leitlinie stellt damit einen Meilenstein in der Standardisierung der CI-Behandlung in Deutschland dar. Auf dieser Grundlage wurden die konsentierten praktischen Umsetzungsempfehlungen unter Federführung der DGHNO-KHC erarbeitet und 2021 als CI-Weißbuch veröffentlicht [3]. Im CI-Weißbuch wurden bereits die inhaltlichen Grundzüge eines deutschlandweiten CI-Registers beschrieben, dessen praktische Umsetzung als DCIR [8] in dieser Arbeit dargelegt wird.

### Entscheidungsprozess zur Einrichtung des DCIR

Parallel zur Erarbeitung der CI-Leitlinie und der Erstellung des CI-Weißbuchs zur Strukturierung des CI-Versorgungsprozesses erfolgte in Deutschland nachfolgend die Einführung eines unabhängigen Zertifizierungsprozesses zur Qualitätssicherung der CI-Versorgung [9]. Bereits im Jahr 2016 hat das Präsidium der DGHNO-KHC nach intensiver Diskussion die grundsätzliche Entscheidung getroffen, wissenschaftlich orientiert an der Weiterentwicklung der Qualitätssicherung der CI-Versorgung mitzuwirken. Hierzu wurde auch die Einrichtung eines nationalen CI-Registers als wesentlich erachtet. Nach entsprechender fachlicher Vorarbeit (CI-Leitlinie und CI-Weißbuch) erfolgte schließlich im November 2021 die Beschlussfassung des Präsidiums der DGHNO-KHC zur Kooperation mit einem externen Registerbetreiber. Nach Abwägung verschiedener potenzieller Anbieter wurde hierfür ein Registerbetreiber mit großer audiologischer Expertise gesucht. Durch das Präsidium der DGHNO-KHC wurde entschieden, das DCIR schließlich in Kooperation mit der Fa. INNOFORCE (Ruggell, Liechtenstein) als Registerbetreiber technisch umzusetzen. Die Umsetzung des DCIR erfolgte unter wissenschaftlicher Leitung des Präsidiums der DGHNO-KHC.

### Organisationsstruktur und Rechtsverhältnisse

Das Präsidium der DGHNO-KHC entwickelte einen Leistungskatalog, auf dessen Basis die Inhalte, die Struktur und der Betrieb des DCIR durch den Registerbetreiber festlegt wurden. Die Kriterien umfassten im Wesentlichen die technische Umsetzung einer Registerdatenbank, inklusive Application-Programming-Interface(API)-Schnittstelle zur Überführung von Daten aus an den Kliniken bereits existierenden Datenbanken, die Entwicklung eines Datenschutzkonzepts und den produktiven Betrieb des DCIR. Auch die Verarbeitung der pseudonymisierten Daten zur Erstellung eines Jahresberichts für jede teilnehmende Klinik sowie die Erstellung eines nationalen Jahresberichts für die DGHNO-KHC gehören zu den vereinbarten Aufgaben des Registerbetreibers.

**Figure 1:**
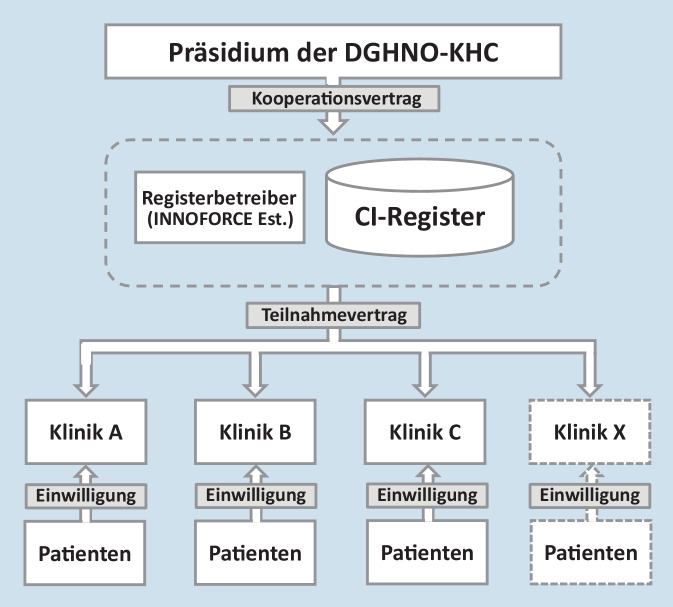

Der Registerbetreiber schließt hierzu als datenschutzrechtlicher Verantwortlicher mit den interessierten Kliniken jeweils einen Teilnahmevertrag am DCIR ab. Hierfür erhält die jeweilige Klinik den Jahresbericht über die jeweils im Kalenderjahr eingegebenen Daten. Es erfolgt ausschließlich die Übermittlung pseudonymisierter Daten an das DCIR. Zu den Aufgaben der teilnehmenden Kliniken gehört die Information der Patienten, deren Daten registriert werden, über die Zielsetzung und das Datenschutzkonzept sowie das Einholen und Dokumentieren der individuellen Patienteneinwilligung zur Datenweitergabe an das DCIR (Abb. 1).

Es besteht keine unmittelbare vertragliche Rechtsbeziehung zwischen dem Betreiber des DCIR und Patienten. Ebenfalls besteht keine unmittelbare Rechtsbeziehung zwischen den teilnehmenden Kliniken und dem Präsidium der DGHNO-KHC in Bezug auf das DCIR. Die wissenschaftliche Leitung des DCIR liegt ebenso beim Präsidium der DGHNO-KHC wie auch die Nutzungsrechte der anonymisierten nationalen Daten.

### Datenschutzkonzept

Die Erfassung von klinischen Daten aus der Patientenbehandlung für das DCIR erfordert, auch bei Verwendung pseudonymisierter Daten, die Einwilligung jedes Patienten. Diese Einwilligung musste daher von der teilnehmenden Klinik für jeden Patienten, dessen Daten im DCIR registriert werden sollten, eingeholt werden. Hierzu wurde eine Mustereinwilligung erarbeitet und den teilnehmenden Kliniken zur Verfügung gestellt.

Die Datenübertragung aus einer teilnehmenden Klinik an das DCIR soll ausschließlich anhand pseudonymisierter Daten erfolgen. Die Identifikation individueller Patienten bzw. deren Daten ist daher nach Übertragung an das DCIR weder für den Registerbetreiber noch für das Präsidium der DGHNO-KHC möglich.

**Figure 2:**
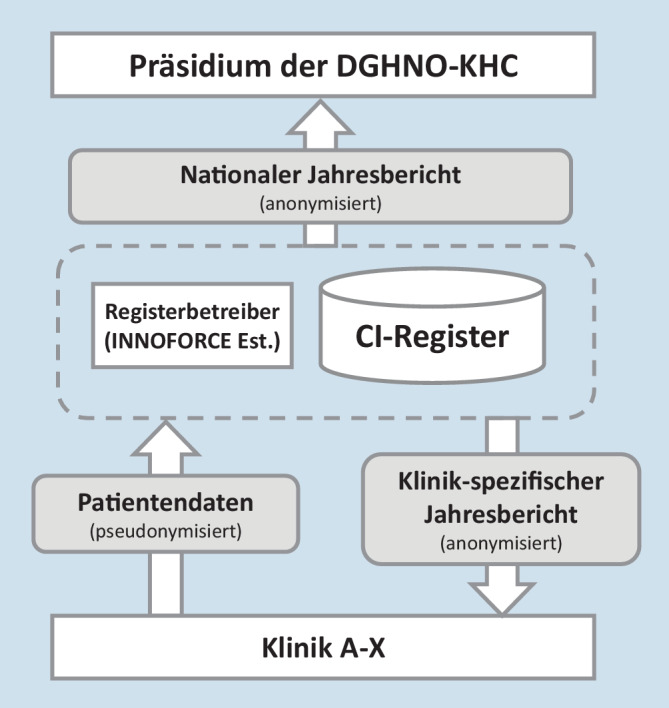

Jede teilnehmende Klinik sollte die von ihr in das Register eingegebenen Daten in Form eines anonymisierten Jahresberichts erhalten. Der Jahresbericht ist damit ein Benchmark, um die jeweiligen Klinikdaten (z. B. Anzahl der Komplikationen) im Vergleich zu den nationalen Gesamtdaten des DCIR zu vergleichen. Das Präsidium der DGHNO-KHC erhält einen anonymisierten nationalen Jahresbericht aller Daten, der keine Rückschlüsse auf einzelne Kliniken oder individuelle Patienten erlaubt (Abb. 2). Das dargestellte Datenschutzkonzept wurde vor der Inbetriebnahme des DCIR sowohl juristisch als auch durch die Datenschutzbeauftragten der jeweiligen Kliniken geprüft.

### Technische Umsetzung der Dateneingabe in das CI-Register

**Figure 3:**
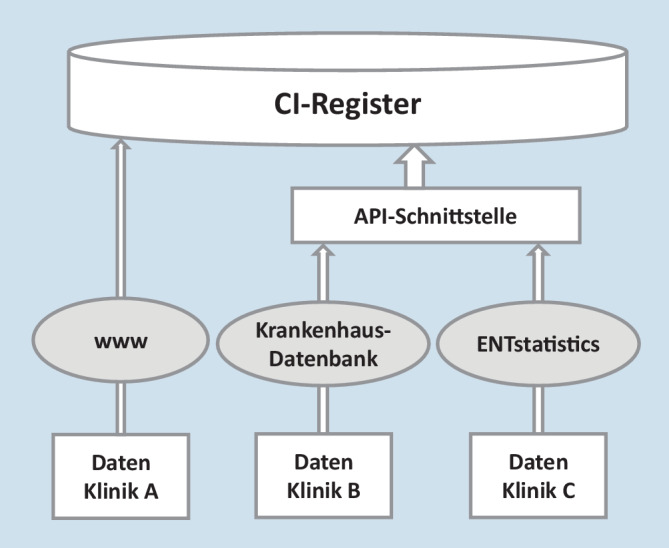

In der Konzeptionsphase des DCIR zeigte sich eine sehr heterogene Ausgangslage in Bezug auf die zur Qualitätssicherung der CI-Versorgung in den Kliniken verwendeten Datenbanken bzw. Dokumentationssysteme. Eine für alle teilnahmewilligen Kliniken umsetzbare Lösung musste daher einerseits die unterschiedlichen Ausgangssituationen berücksichtigen und anderseits eine homogene Datenqualität des DCIR sicherstellen. Infolgedessen wurden verschiedene technische Zugangsmöglichkeiten zur Datenübertragung erarbeitet und den Kliniken individuell zur Nutzung angeboten. Diese umfassten entweder 1. die internetbasierte Dateneingabe, 2. die Verwendung einer bereits existierenden Datenbank oder 3. die Einrichtung einer Datenbank des Registerbetreibers (Abb. 3). Sowohl die Verwendung einer Krankhausdatenbank als auch von ENTstatistics (Datenbank des Registerbetreibers) ermöglichen den automatisierten Datenimport aus audiometrischen Endgeräten.

#### Internetbasierte Dateneingabe

Für Kliniken, die entweder bislang kein eigenes EDV-System zur Dokumentation CI-bezogener Daten betrieben haben oder nur wenige CI-Fälle pro Jahr versorgen, sollte die Möglichkeit einer direkten internetbasierten Dateneingabe für das DCIR geschaffen werden. Hierzu wurde vom Registerbetreiber ein internetbasierter Registerzugang entwickelt, der online eine „händische“ Eingabe der Registerdaten ermöglicht. Dieser Zugang erlaubt damit einer Klinik auch ohne weitere technische Voraussetzungen, wie die Einrichtung einer separaten Krankenhausdatenbank, eine Teilnahme am DCIR.

#### Übertragung von Daten aus einer existierenden Krankenhausdatenbank

Eine große Anzahl der am DCIR teilnehmenden Kliniken betreiben bereits eigene Krankenhausdatenbanken oder Dokumentationssysteme zur Qualitätssicherung der CI-Versorgung. Die meisten dieser lokalen Datenbanken können periphere Endgeräte (z. B. Audiometer) anbinden und deren Befunde importieren. Um eine doppelte Datenerfassung zu vermeiden, musste die Möglichkeit geschaffen werden, die lokal erfassten CI-Therapiedaten an das Register zu übermitteln. Hierfür wird durch den Registerbetreiber seit Herbst 2022 eine API-Schnittstelle bereitgestellt.

#### Verwendung der Datenbank des Registerbetreibers

Der Registerbetreiber (Fa. INNOFORCE, Ruggell, Liechtenstein) verfügt über eine HNO-Datenbank (ENTstatistics) [10]. Dieses System wird von vielen Kliniken verwendet, um otologische, rhinologische, laryngologische und Tumorbefunde zu dokumentieren und statistisch auszuwerten. ENTstatistics bietet insbesondere Schnittstellen zur Integration peripherer Endgeräte an. Das System unterstützt die Dokumentation der für das DCIR erforderlichen Therapiedaten sowie die anschließende Übertragung ans DCIR.

### Inhalt des DCIR

Das DCIR orientiert sich primär an der Dokumentation des Implantats bzw. der Implantation. Damit werden ausschließlich Patienten in das Register aufgenommen, die tatsächlich ein Implantat erhalten haben. Das Register ist rein prospektiv angelegt, sodass erst ab dem Zeitpunkt der Betriebsaufnahme des Registers (Januar 2022) Implantationen und Implantate registriert werden konnten. Die Registersystematik beruht daher auf der Erfassung und Dokumentation der zur Beurteilung der Implantatfunktion relevanten Parameter. Diese sind in 10 „Datenblöcke“ unterteilt, die sich an der aktuellen CI-Leitlinie [2] orientieren. Diese umfassen im Einzelnen: Basisdaten, präoperative Audiometrie, präoperative Höranamnese, Implantat, Operation, CI-bezogene Komplikationen, CI-Nutzung und Rehabilitationsfortschritt, postoperative Audiometrie, Hör-/Sprachentwicklung (Kinder) und die Lebensqualität. Darüber hinaus beinhalten die Datenblöcke aber auch die Erfassung einer leitlinienkonformen CI-Versorgung. Dieser Behandlungsprozess umfasst sowohl die präoperative Phase, die operative Phase, die Basistherapie, die Folgetherapie als auch die lebenslange Nachsorge. Die Definition und der Inhalt der Datenblöcke wurde bereits in die aktuelle Fassung des CI-Weißbuchs der DGHNO-KHC integriert [3]. Eine Übersichtsdarstellung über die Datenblöcke sowie deren Inhalte findet sich in Tab. 1 sowie die vollständige Auflistung aller erhobenen Registerparameter im beigefügten Supplement.

**Table 1:** 

*1. Basisdaten*	*6. CI-bezogene Komplikation*
ID (Code) Versorgende Einrichtung	Revisionsbedürftige Fehllage der Elektrode
Patienten-ID, Pseudonym	Fazialisparese
Geburts‑,Sterbedatum; Geschlecht; Muttersprache	Stationäre Aufnahme wg. CI-bezogener Komplikation
*2. Präoperative Audiometrie*	Meningitis nach CI-Versorgung
Tonaudiogramm	Tod in Verbindung mit der CI-Versorgung
Sprachtest (z. B. Freiburger Einsilbertest); Satztest (z. B. OlSa)	*7. CI-Nutzung und Rehabilitation*
Objektive Messung (OAE, BERA, ASSR)	Implantatfunktion
*3. Präoperative Höranamnese*	Nutzungsdauer
Zeitpunkt des Hörverlust	Aktueller Rehabilitationsstatus
Hörverlust/Taubheit in Jahren	*8. Postoperative Audiometrie*
HG-Nutzung im zu versorgenden Ohr	Zeit nach CI-Operation
Versorgung Gegenohr	Tonaudiogramm; Sprachtest; Satztest
Art und Ursache der Hörstörung	*9. Hör‑/Sprachentwicklung Kinder*
*4. Implantat*	Verwendung alternativer Kommunikation
Implantationsdatum	Auditive Wahrnehmungsentwicklung
Implantathersteller	Kommunikative Entwicklung
Implantatbezeichnung	Fortschritte der Hör‑/Sprachentwicklung
Implantatseriennummer	Sinnesspezifische Förderung
Explantation (Datum, Grund)	Pädagogische Einrichtung/Schule
*5. Operation*	*10. Lebensqualität*
Operationsdatum und Operationsgrund	Fragebogen zur Lebensqualität
Elektrodeninsertion/Insertionstiefe	
Intraoperative Funktionskontrolle	
Durchführung radiologische Lagekontrolle Elektrode	
Revisionsoperation	

Die vollständige Auflistung der erhobenen Daten findet sich im CI-Weißbuch der DGHNO-KHC [3] und als Supplement (ESM-Material)

*ASSR *„auditory steady state response“; *BERA* „brainstem-evoked response audiometry“; *CI* Cochlea-Implantat; *DGHNO-KHC *Deutsche Gesellschaft für Hals-Nasen-Ohren-Heilkunde, Kopf- und Hals-Chirurgie; *HG *Hörgerät; *ID* Identifikationsmerkmal; *OAE* otoakustische Emissionen; *OlSa *Oldenburger Satztest

### Zeitpunkte der Datenerhebung

**Figure 4:**
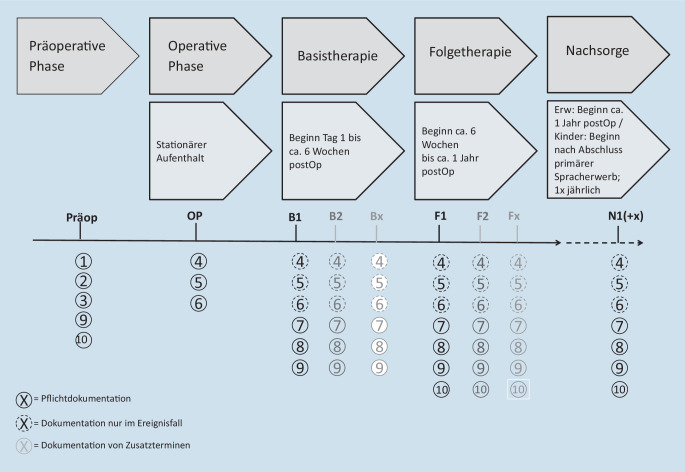

Die Zeitpunkte der Dokumentation einzelner Datenblöcke orientiert sich ebenfalls an dem in der CI-Leitlinie und dem CI-Weißbuch konsentierten Behandlungsprozess, der sich in 5 Phasen unterteilt (präoperative Phase, operative Phase, Basistherapie, Folgetherapie, Nachsorge; Abb. 4). Auch wenn zahlreiche individuelle, krankenhausspezifische Behandlungskonzepte existieren, die den zeitlichen Umfang der einzelnen Phasen variabel gestalten, besteht dennoch wissenschaftlicher Konsens in der prinzipiellen zeitlichen Zuordnung dieser Abschnitte (Abb. 4). Das DCIR sieht daher vor, zu jeder der einzelnen Phasen mindestens einen Zeitpunkt zur Datenerhebung zu dokumentieren, um alle Phasen der Hörrehabilitation mit CI abzubilden. Da einzelne Phasen, z. B. die Folgetherapie, patientenindividuell unterschiedlich viele Einzeltermine aufweisen können, kann die Anzahl der Dateneinträge deutlich variieren.

Wie in Abb. 4 dargestellt, orientiert sich die Struktur der Datenerhebung am Versorgungsprozess gemäß CI-Leitlinie [2]. Die in der jeweiligen Phase zu erhebenden Datenblöcke sind mit den Nummern 1–10 bezeichnet (Tab. 1). Die angegebenen Ziffern für die jeweilige Versorgungsphase (z. B. B1, B2, BX) beschreiben den ersten, zweiten oder x‑ten Kontakt der Klinik mit dem Patienten während der jeweiligen Phase (im Beispiel aus Abb. 4: B = Basistherapie). Analog hierzu erfolgen die Bezeichnung und Nummerierung in der Folgetherapie und der Nachsorge.

Das DCIR ermöglicht prinzipiell, für jede Versorgungsphase beliebig viele Zeitpunkte zur Dateneinabe zu dokumentieren. Mindestens muss allerdings ein Eintrag für jede einzelne Phase erfolgen. Als zeitliche Orientierung kann für die Versorgungsphasen bei Erwachsenen die Basistherapie bis etwa 6 Wochen postoperativ angenommen werden, für die Folgetherapie bis etwa ein Jahr postoperativ, und für die Nachsorge beginnt der Zeitraum etwa ein Jahr postoperativ. Individuell sind hiervon allerdings abweichende Zeiträume möglich. Für Kinder sind ebenfalls hiervon deutlich abweichende Zeiträume anzusetzen. Die Grundzüge der Datenerhebungszeitpunkte wurden bereits in der aktuellen Fassung des CI-Weißbuchs der DGHNO-KHC beschrieben [3].

Die Struktur des DCIR sieht eine Mindestdokumentation für einzelne Datenblöcke in jeder Phase des Versorgungsprozesses vor. Im Gegensatz hierzu werden andere Datenblöcke (z. B. Datenblock 6: Komplikationen) nur im Ereignisfall dokumentiert. Dieses Vorgehen ermöglicht einen praktikablen Weg zwischen Dokumentationsumfang und umsetzbarem Aufwand für die teilnehmende Klinik. Eine Übersichtsdarstellung der Pflicht- und Ereignisdokumentation findet sich in Abb. 4. Ebenfalls wird hier ein Anhaltspunkt für die Zeiträume der jeweiligen Phase gegeben.

### Datenauswertung und Erstellung der Jahresberichte

**Figure 5:**
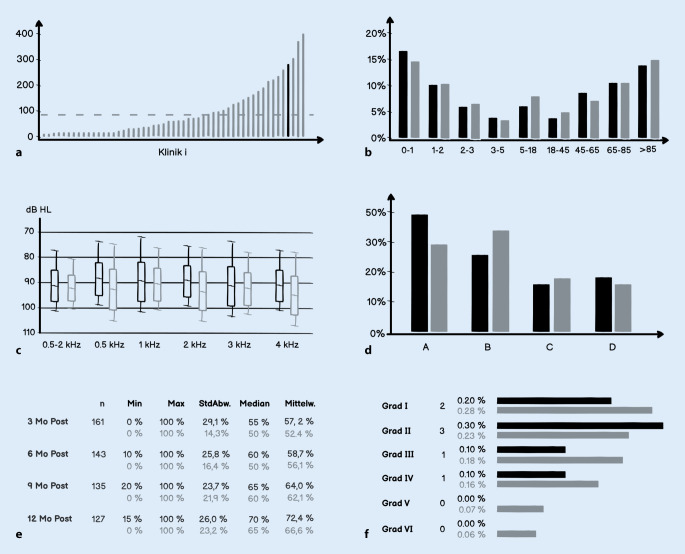

Der Registerbetreiber erstellt für jede teilnehmende Klinik einen anonymisierten Jahresbericht auf der Grundlage der durch die Klinik eingegebenen Daten (Abb. 2). Diese Daten werden im Vergleich zu den nationalen Gesamtdaten präsentiert, sodass ein relativer Vergleich (Benchmarking) für die jeweilige Klinik ermöglicht wird. Klinikeigene Daten können daher im Vergleich zu den deutschlandweiten Durchschnittsdaten betrachtet werden. Eine modellhafte Darstellung von Auszügen aus einem klinikspezifischen Jahresbericht ist in Abb. 5 dargestellt.

Der Registerbetreiber stellt dem Präsidium der DGHNO-KHC zusätzlich einen anonymisierten Jahresbericht über alle in das Register eingegebenen Daten zur Verfügung. Eine Identifikation einzelner Patienten oder einzelner Kliniken in Bezug zu den bereitgestellten Daten ist im nationalen Jahresbericht nicht möglich. Kliniken werden hier lediglich anonymisiert aufgeführt, sodass nur anonymisierte Klinikvergleiche ermöglicht werden. Lediglich dem Registerbetreiber ist die Identität der Klinik bekannt, um im Fall schwerwiegender Auffälligkeiten eine Rückmeldung an die Einrichtung zu geben.

### Entwicklung eines Logos für das DCIR

**Figure 6:**
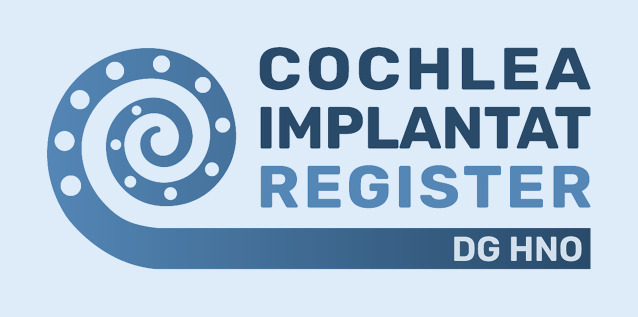

Um eine Erkennbarkeit der auf der Grundlage des DCIR erhobenen Daten und Veröffentlichungen zu ermöglichen, wurde in Zusammenarbeit des Präsidiums der DGHNO-KHC mit dem Registerbetreiber ein Registerlogo entwickelt, das allen teilnehmenden Partnern des DCIR zur internen und externen Kommunikation zur Verfügung gestellt wird (Abb. 6).

## Ergebnisse

### Einrichtung und Betrieb des DCIR

Der produktive Betrieb des DCIR mit der browserbasierten Dateneingabe wurde im Januar 2022 aufgenommen. Seit Januar 2022 können pseudonymisierte Daten in das DCIR eingegeben werden. In den ersten 15 Monaten des Registerbetriebs wurden bereits mehr als 2500 CI von mehr als 2000 Patienten erfolgreich in das DCIR aufgenommen. Anzumerken ist hierbei, dass die Anzahl der Implantate nicht der Anzahl der Patienten entspricht, da Patienten auch bilateral CI-versorgt sein können. Aktuell erfolgt die detaillierte Datenanalyse, sodass eine inhaltliche Darstellung der erhobenen Ergebnisse in einer separaten wissenschaftlichen Auswertung erfolgen wird.

### Automatisierter Datenexport

Die 3 unterschiedlichen Mechanismen zur Datenerfassung (Abb. 3) wurden zwischenzeitlich umgesetzt. Die API-Schnittstelle ist seit Herbst 2022 verfügbar. Hierdurch ließen sich erfolgreich individuelle Lösungen für die teilnehmenden Kliniken finden, um eine Registerteilnahme zu gewährleisten.

### Teilnehmende Kliniken

Nach Fertigstellung des Leistungskatalogs und dem anschließenden Projektstart konnten Kliniken seit Juli 2021 ihre Bereitschaft zur Registerteilnahme erklären. Den Teilnahmevertrag zum DCIR schlossen 11 Kliniken im Jahr 2021 und 64 Kliniken im Jahr 2022 ab. Zum Zeitpunkt März 2023 hatten damit insgesamt 75 Kliniken vertraglich ihre Teilnahme am DCIR erklärt.

### Jahresberichte 2022

Die Dateneingabe in das DCIR für das Jahr 2022 wurde zwischenzeitlich für die teilnehmenden Kliniken erfolgreich abgeschlossen. Gegenwärtig erfolgt der Auswertungsprozess, sodass sowohl die Fertigstellung der Jahresberichte 2022 für die teilnehmenden Kliniken als auch die Erstellung des nationalen Jahresberichts 2022 absehbar in den nächsten Wochen erfolgen werden. Eine Auswertung und wissenschaftliche Veröffentlichung zu den Inhalten des nationalen Jahresberichts befindet sich gegenwärtig ebenfalls durch das Präsidium der DGHNO-KHC in Vorbereitung.

### Öffentliche Verfügbarkeit von Informationen zum DCIR

Mit der technischen Umsetzung des DCIR ging die öffentliche Bereitstellung von Informationen über die Zielsetzung und die Inhalte des Registers einher. Hierzu wurde vom Betreiber des Registers eine Internetseite mit freiem Zugang eingerichtet (https://www.ci-register.de/).

## Diskussion

Die Entwicklung, Strukturierung und der Betrieb eines nationalen klinischen Registers ist eine komplexe Aufgabe, die erhebliche zeitliche und finanzielle Ressourcen erfordert. Sämtliche Entwicklungsschritte zum inzwischen erfolgreichen Betrieb des DCIR wurden eigeninitiativ durch die DGHNO-KHC und den Registerbetreiber realisiert. Neben der Definition der Registerinhalte erfolgte die erfolgreiche Erarbeitung der rechtlichen und vertraglichen Grundlagen zur Einrichtung und zum Betrieb sowie Entwicklung von Jahresberichten und eines Logos. Der produktive Betrieb des DCIR wurde Anfang 2022 aufgenommen. Seit der Inbetriebnahme wurden damit in etwas mehr als einem Jahr bereits mehr als 2500 Implantate von mehr als 2000 Patienten in das DCIR aufgenommen. Perspektivisch ist damit klar erkennbar, dass innerhalb kurzer Zeit die Datenbasis des DCIR ein sehr breites Fundament haben wird, um sowohl wissenschaftliche Fragestellungen zu beantworten als auch zukünftige Qualitätsstandards hieraus abzuleiten. Die aus dem DCIR gewonnenen Erkenntnisse werden damit zukünftig einen wesentlichen Beitrag zur Qualitätssicherung und Weiterentwicklung wissenschaftlich basierter Qualitätsstandards in der CI-Versorgung leisten.

### Teilnahmevoraussetzungen

Die Teilnahme am DCIR ist grundsätzlich für alle Kliniken möglich, die den vertraglichen Voraussetzungen mit dem Registerbetreiber zustimmen und die technischen Voraussetzungen zur Dateneingabe erfüllen. Bereits in der kurzen Zeit seit der Inbetriebnahme des DCIR haben 75 Kliniken vertraglich ihre Mitarbeit im CI-Register zugesagt. Dies ist ein beeindruckendes Ergebnis, welches das große Interesse der Kliniken an einer Registerteilnahme illustriert. Im Gegenzug erhalten die teilnehmenden Kliniken einen Jahresbericht, der ein Benchmarking der Klinikdaten im Vergleich zu den nationalen Durchschnittsdaten erlaubt. Dieser Bericht kann für die Klinik auch unmittelbar als Qualitätsbericht Verwendung finden. Eine teilnehmende Klinik liefert damit nicht nur einen wichtigen aktiven Beitrag zur Weiterentwicklung neuer Qualitätsstandards, sondern sie erhält auch einen unmittelbaren Nutzen in Form einer standardisierten und professionellen Aufarbeitung ihrer Daten für einen CI-Qualitätsbericht. Ein CI-Qualitätsbericht ist zudem nach der aktuellen CI-Leitlinie und dem CI-Weißbuch gefordert, um auch für Patienten eine transparente Angabe zur Versorgungsqualität einer Einrichtung einfach verfügbar zu machen [2, 3].

Die Bereitstellung der technischen Voraussetzungen zur Datenübertragung aus einer Klinik stellte eine besondere Herausforderung in der Umsetzung des DCIR dar. Um eine möglichst hohe Teilnahme am DCIR zu erreichen, mussten daher unterschiedliche technische Lösungen entwickelt werden. Dies schloss auch die Option einer browserbasierten Einzeldateneingabe ein. Für die Nutzung der API-Schnittstelle zur Übermittlung von Daten aus lokalen Datenbanken entstehen den Kliniken keine zusätzlichen Kosten seitens des DCIR. Die API-Funktionalität ist in den Grundfunktionen des Registers bereits enthalten. Allerdings können einer Klinik Aufwände für die Realisierung (z. B. Programmierung) des Exports aus der lokalen Datenbank entstehen. Einige Kliniken haben anlässlich des Teilnahmewunsches am DCIR von der Exportmöglichkeit profitiert und ihre eigene Datenbank adaptiert bzw. eine neue lokale Datenbank, z. B. ENTstatistics (Fa. INNOFORCE, Ruggell, Liechtenstein), implementiert. Der Registerbetreiber hat den Kliniken hierzu individuelle, auf die jeweiligen Bedürfnisse zugeschnittene Lösungen angeboten.

Bereits im ersten Jahr nach Registereinrichtung konnten auf diese Weise erfolgreiche Datenexporte einzelner teilnehmender Kliniken durchgeführt werden. Da sowohl die finanzielle Ausstattung als auch die Investitionsmöglichkeiten zwischen den Kliniken erheblich divergieren, wurde diesem Aspekt in der Konzeptionierung des Registers mit unterschiedlichen Zugangsmöglichkeiten Rechnung getragen. Durch dieses Konzept konnten Kliniken selbst entscheiden, welchen Automatisierungsgrad der Datenübertragung (browserbasierte Eingabe, Übertragung aus existierender Datenbank oder neu einzurichtende Datenbank) sie umsetzen möchten. Durch die im ersten Jahr gesammelten Erfahrungen wurde die grundsätzliche Nutzbarkeit aller 3 dargestellten Dateneingabemöglichkeiten für das DCIR belegt.

### Beurteilung der Versorgungsqualität

Ein besonderer Wert eines klinischen Registers liegt in der langfristigen Beurteilung der Versorgungsqualität. Diese umfasst nicht nur akute Komplikationen, sondern erhebt auch die langfristige Ergebnisqualität eines Therapieverfahrens. Gerade der Skandal um die fehlerhaften Brustimplantate [11] illustriert diesen Aspekt eindrücklich. In der Folge wurden inzwischen in Deutschland durch das Implantateregistergesetz (IRegG) [7] die rechtlichen Grundlagen geschaffen, um eine Vielzahl von medizinischen Implantaten verpflichtend in einem Register zu dokumentieren. Auch das CI ist in diesem Gesetz ausdrücklich genannt, sodass in Zukunft nicht nur eine freiwillige und rein wissenschaftlich-orientierte Dokumentation der CI-Versorgung erfolgen wird, sondern eine Dokumentation gesetzlich verpflichtend sein wird. Unklar bleibt gegenwärtig, warum neben CI nicht auch andere implantierbare Hörsysteme, wie z. B. aktive Mittelohrimplantate, in das IRegG aufgenommen wurden. Auch wenn der zeitliche Beginn einer Pflichtdokumentation der CI gegenwärtig nicht exakt bekannt ist, hat die DGHNO-KHC mit der hier präsentierten Registerinitiative bereits den fachlichen Standard gesetzt und auch einen Dialog mit den staatlichen Registerstellen begonnen (Bundesinstitut für Arzneimittel und Medizinprodukte, BfArM). Durch die Einrichtung und den Betrieb des DCIR wurden fachspezifische Parameter frühzeitig definiert und deren Umsetzung seit Anfang 2022 praktisch umgesetzt. Diese Initiative kann daher als beispielgebend für andere medizinische Implantate oder auch andere Fachgesellschaften betrachtet werden.

Zusätzlich ist 2021 eine neue EU-Verordnung für Medizinprodukte in Kraft getreten, die die Implantathersteller verpflichtet, u. a. auch klinische Performance-Daten ihrer Implantate in einer langjährigen Nachverfolgung bereitzustellen. Diese als Medical Device Regulation (MDR) bezeichnete Verordnung [12] stellt Kliniken und die Hersteller der Implantate vor erhebliche Herausforderungen. Es bleibt abzuwarten, ob das DCIR auch in Bezug auf die MDR einen Beitrag leisten kann.

### Akzeptanz

CI-versorgende Einrichtungen (CIVE), die auf der Basis der CI-Leitlinie die notwendige Strukturqualität, Prozessqualität und Ergebnisqualität aufweisen, können sich seit 2021 in einem strukturierten Prozess zertifizieren lassen [7]. Deutschlandweit haben inzwischen bereits 47 Kliniken das CIVE-Zertifikat erlangt [13]. Eine Voraussetzung zur erfolgreichen Zertifizierung ist die Verpflichtung der Kliniken, aktiv am DCIR teilzunehmen. Ohne eine entsprechende Verpflichtung kann das CIVE-Zertifikat einer Klinik nicht erteilt werden. Betrachtet man die Anzahl der Kliniken, die sich verpflichtet haben, allein am DCIR, nicht aber an der Zertifizierung teilzunehmen (75 Kliniken), ergibt sich hier eine deutliche Differenz zwischen zertifizierten CI-versorgenden Einrichtungen und ausschließlich am Register teilnehmenden Kliniken. Diese Differenz könnte aus unterschiedlichen Perspektiven erklärt werden. Zum einen könnten Kliniken, die möglicherweise zum gegenwärtigen Zeitpunkt noch nicht sämtliche Bedingungen für eine erfolgreiche Zertifizierung erfüllen, dennoch entschieden haben, bereits am Register teilnehmen zu wollen. Möglicherweise beantragen diese Kliniken die Erteilung des CIVE-Zertifikats zu einem späteren Zeitpunkt. Eine weitere Erklärung könnte darin bestehen, dass Kliniken keine Zertifizierung anstreben, aber die Vorteile der Registerteilnahme in Anspruch nehmen möchten. Da ein strukturierter Jahresbericht als Nutzen für diese Kliniken resultiert, könnte das DCIR für diese Kliniken als eine reine Datenbank genutzt werden, da auch diese Kliniken die von ihnen in das Register eingegebenen pseudonymisierten Rohdaten am Jahresende gesammelt zurückerhalten. Insofern ergeben sich für Kliniken aus einer Registerteilnahme vielfältige Vorteile, die bereits ab dem ersten Jahr der Registerteilnahme bestehen.

Eine weitere Erklärung für die hohe Anzahl der am Register teilnehmenden Kliniken könnte auch in der Preisgestaltung des Registerbetreibers liegen. Hier bestand in den ersten Monaten des Beitritts am Register ein finanziell besonders attraktives Angebot.

Seit der Inbetriebnahme des DCIR haben bereits 75 Kliniken vertraglich ihre Teilnahme an dem Register zugesagt. Dies ist eine, für diesen kurzen Zeitraum, sehr positive Entwicklung, besonders vor dem Hintergrund der prinzipiell verfügbaren Kliniken, die am Register teilnehmen könnten. Die exakte Anzahl der Einrichtungen, die eine CI-Versorgung vornehmen, ist für Deutschland nicht abschließend bekannt. In einer 2020 durch die DGHNO-KHC in Zusammenarbeit mit der Patientenselbsthilfe (Deutsche Cochlea Implantat Gesellschaft e. V., DCIG) durchgeführten Untersuchung gaben 70 von 170 in Deutschland existierenden HNO-Kliniken an, eine CI-Versorgung durchzuführen. Einschränkend muss angemerkt werden, dass die Anzahl der an dieser Untersuchung teilnehmenden Kliniken nicht vollständig war, sodass die Autoren eher von einer Anzahl von etwa 100 Kliniken ausgehen, die eine CI-Operation anbieten [14]. Vor diesem Hintergrund ist eine Anzahl von 75 Kliniken, die ihre Teilnahme am Register zugesagt haben, noch beeindruckender, da sie offensichtlich bereits der Mehrheit der CI-Kliniken in Deutschland entspricht. Diese hohe Teilnehmerzahl belegt nicht nur das große Interesse der Kliniken zur aktiven Teilnahme am Register, sondern zeigt auch die zukünftig zu erwartende repräsentative Abdeckung der in Deutschland vorhandenen klinischen Daten durch das DCIR.

### Vergleich mit anderen Registern

In Deutschland ist bereits in der Vergangenheit eine Vielzahl klinischer Register sehr erfolgreich eingeführt worden, um Versorgungsparameter zu erheben. Beispielhaft kann hier das Traumaregister der Akademie der Unfallchirurgie (AUC) genannt werden [5]. Dieses Register wird bereits seit 1993 betrieben und kann als wegweisend für medizinisch-wissenschaftliche Register betrachtet werden, die primär auf die Verbesserung der Versorgungsqualität ausgerichtet sind. Die Zielsetzung des CI-Registers ähnelt daher bereits etablierten Konzepten, da durch den wissenschaftlichen Ansatz, getragen durch die Fachgesellschaft (DGHNO-KHC), nicht kommerzielle Interessen, sondern fachliche, wissenschaftliche und qualitätsorientierte Interessen die Registereinrichtung motivieren. Bezogen auf die erhobenen Daten stand daher außer Frage, dass ausschließlich eine anonymisierte Datenanalyse erfolgen würde. Um ein realistisches Benchmarking zu ermöglichen, muss auf der anderen Seite allerdings ein Vergleich der jeweiligen Klinikdaten zu den nationalen Durchschnittsdaten möglich sein. Das hier präsentierte Auswertungssystem verbindet die Klinikinteressen (anonymes Benchmarking) mit den Interessen der Fachgesellschaft zur Erstellung eines nationalen Jahresberichts. Erstmals wird durch diesen Ansatz eine sehr große Anzahl von Versorgungsprozessen und versorgten Patienten deutschlandweit erhoben.

### Internationaler Vergleich

Im internationalen Vergleich existieren bereits einige CI-Register. Beispielhaft können hier das Schweizer und das französische CI-Register genannt werden [15, 16]. In der Schweiz wurde das Register bereits im Jahr 1992 eingeführt und seitdem kontinuierlich betrieben. In einer kürzlich erschienenen Arbeit [17] wurde hierzu für Europa gezeigt, dass 2021 erst in 4 Ländern CI-Register etabliert waren (etwa 10 % der Länder Europas). Diesbezüglich stellt die Einführung des DCIR nicht den ersten Ansatz zur Qualitätssicherung der CI-Versorgung in einem europäischen Land dar. Berücksichtigt man aber die erhobenen Parameter in anderen europäischen CI-Registern, erscheint der hier präsentierte Ansatz des DCIR, über 10 definierte Datenblöcke entsprechende Parameter zu erheben, einzigartig und innovativ. Wesentliche Unterschiede des DCIR zu anderen Registern bestehen insbesondere in der Erfassung des gesamten CI-Versorgungsprozesses und der lebenslangen Nachsorge, die bedeutende Qualitätsparameter darstellen.

### Limitationen

Trotz einer bisherigen sehr positiven Entwicklung des DCIR unterliegt der methodische Ansatz dennoch einigen Limitationen, die im Folgenden erörtert werden.

Die wesentliche Herausforderung in der langfristigen Sicherung des Betriebs des Registers besteht in der möglichst vollständigen Erfassung der Daten von CI-Patienten. Gegenwärtig ist die Teilnahme am Register für Kliniken eine rein freiwillige Leistung, die motiviert ist durch den Wunsch zur weiteren Verbesserung der CI-Versorgungsqualität, der zukünftigen Erarbeitung neuer Qualitätsstandards und auch der Bereitstellung eines Jahresberichts für teilnehmende Kliniken. Eine Verpflichtung zur Bereitstellung der Daten existiert gegenwärtig nicht. Als Orientierungspunkt zur Beurteilung der Abdeckung der verfügbaren Daten durch das Register kann die Anzahl der durchgeführten Operationen anhand der durch das Statistische Bundesamt bereitgestellten Diagnosis-Related-Groups(DRG)-Statistik genutzt werden. Hiernach fanden im Jahr 2021 in Deutschland 4359 CI-Operationen [18] statt. Berücksichtigt man die im Vergleich hierzu in etwa 15 Monaten bereits in das DCIR aufgenommene Anzahl der Implantate (Stand März 2023: > 2500 Implantate), scheint bereits zum jetzigen Zeitpunkt die Durchdringung des Registers für die CI-Versorgung in Deutschland sehr hoch: Etwa 50 % der Implantationen sind demnach, unter der Annahme annähernd gleicher Versorgungszahlen 2021 und 2022, bereits in der kurzen Betriebszeit des DCIR in das Register aufgenommen worden. Auch wenn dies in der Anfangsphase des Registers ein bemerkenswerter Erfolg ist, muss kritisch angemerkt werden, dass etwa 50 % der Implantationen gegenwärtig noch nicht dokumentiert werden. Ob dies an noch nicht behobenen individuellen technischen Schwierigkeiten, einer inkompletten Datenübertragung oder an nicht teilnahmewilligen Kliniken liegt, kann gegenwärtig nicht abschließend beurteilt werden. Spätestens mit der zukünftigen Umsetzung des IRegG und dessen Anwendung auf die CI wird hier eine komplette, weil verpflichtende, Dokumentation aller Implantationen absehbar sein. Das DCIR leistet auch hierzu wichtige fachliche Vorarbeiten.

Die Sicherstellung der Datenqualität wird eine wesentliche Bedingung für die wissenschaftliche Nutzbarkeit des Registers darstellen. Dies betrifft sowohl die Vollständigkeit der übermittelten Datensätze als auch besonders die inhaltliche Plausibilisierung der erfassten Daten. Der Qualitätssicherung des DCIR kommt damit zukünftig ebenfalls eine wichtige Bedeutung zu.

Zum gegenwärtigen Zeitpunkt erfordert die Datenübertragung an das Register die Einholung der Einverständniserklärung des jeweiligen Patienten. Es handelt sich hierbei um einen aufwendigen Prozess, und es besteht auch die Möglichkeit, dass ein Patient seine Einwilligung zum Datentransfer nicht erteilt. Momentan liegt der administrative Aufwand auf der Seite der teilnehmenden Kliniken. Zu hoffen bleibt, dass CI-versorgte Patienten auch auf Grundlage der zu erwartenden Ergebnisse und der langfristigen Einflussnahme auf zukünftige Qualitätsstandards hierzu weiter ihre Einwilligung geben, um das DCIR zu unterstützen. Eine entsprechende Präsentation des DCIR und der hieraus resultierenden Daten sollte daher auch CI-versorgten Patienten zugänglich gemacht werden, um eine entsprechende Unterstützung sowohl bei Patienten als auch Patientenselbsthilfeorganisationen zu erreichen. Mit der zukünftigen Einführung des IRegG [7] wird die Dokumentation der erhobenen Daten dann gesetzlich verpflichtend und erfordert keine aktive Einwilligung eines Patienten mehr.

Die Struktur des CI-Registers orientiert sich an den Inhalten der CI-Leitlinie und des CI-Weißbuchs. Der hier dargestellte Versorgungsprozess ist prinzipiell sowohl für Erwachsene als auch für Kinder anzuwenden. Es ist aber offensichtlich, dass neben einer hohen Deckungsgleichheit der zu erhebenden Parameter (z. B. technische Daten oder demographische Daten) für eine Vielzahl der erhobenen Variablen auch altersspezifische Parameter erhoben werden müssen. Gerade im Hinblick auf die Erfolgsbeurteilung ergeben sich hier eine Vielzahl von Herausforderungen, die eine unmittelbare Vergleichbarkeit der Kinder- und Erwachsenendaten erschweren. Gegenwärtig entspricht die Registerstruktur den konsentierten Inhalten der CI-Leitlinie und des CI-Weißbuchs. Es bleibt abzuwarten, ob eine Veränderung der Datenblöcke oder Datenfelder mit der zukünftigen Aktualisierung der CI-Leitlinie bzw. des CI-Weißbuchs erforderlich wird.

### Kosten und Refinanzierung

Die aus dem Register zu erwartende Weiterentwicklung der Qualität der CI-Versorgung liegt nicht nur im Interesse der Patienten und der Kliniken, sondern auch im unmittelbaren Interesse der Kostenträger. Auch zukünftig ist mit wiederkehrenden Kosten für den Betrieb des Registers sowohl seitens der teilnehmenden Kliniken als auch seitens der DGHNO-KHC zu rechnen. Es ist daher offensichtlich, dass die dargestellte Initiative die Unterstützung der Kostenträger finden muss, um auch einen langfristigen Betrieb des DCIR sicherzustellen. Dies ist insbesondere von hoher Bedeutung, da gerade erst in der langjährigen Nachverfolgung der Implantatsicherheit, möglicher Komplikationen, aber auch der Ergebnisqualität der CI-Versorgung ein enormes Potenzial zur wissenschaftlich-basierten Qualitätssicherung besteht. Die Erarbeitung der Grundlagen, die Strukturierung und die Einführung des DCIR bis zur erfolgreichen Inbetriebnahme ist ausschließlich durch Eigeninitiative der DGHNO-KHC sowie der teilnehmenden Kliniken realisiert worden. Die hierzu seitens der DGHNO-KHC erforderlichen Investitionen für die Einrichtung des Registers stellen für die Fachgesellschaft eine erhebliche finanzielle Belastung dar. Auch die teilnehmenden Kliniken tragen Kosten. Sowohl für die Fachgesellschaft als auch für die Kliniken stellt daher das DCIR eine relevante finanzielle Investition dar. Außer Frage steht, dass eine Refinanzierung des Registers, bzw. der langfristige Betrieb, die finanzielle Unterstützung der Kostenträger erforderlich macht.

## Fazit für die Praxis

- Die hier präsentierte Arbeit beschreibt die Strukturierung, den Aufbau und die erfolgreiche Einrichtung des Deutschen Cochlea-Implantat-Registers (DCIR).
- Durch die Umsetzung der in der Cochlea-Implantat(CI)-Leitlinie und im CI-Weißbuch erfolgten Vorarbeiten in Bezug auf struktur-, prozess- und ergebnisqualitätsrelevante Parameter konnte eine konsistente Überführung dieser Inhalte in das DCIR erfolgen.
- Nach der Einführung der Zertifizierung für CI-versorgende Einrichtungen stellt die Einführung des DCIR eine weitere wesentliche Säule zur zukünftigen wissenschaftlich basierten Qualitätssicherung der CI-Versorgung in Deutschland dar.
- Durch die hier beschriebene Initiative der Deutschen Gesellschaft für Hals-Nasen-Ohren-Heilkunde, Kopf- und Hals-Chirurgie e. V. (DGHNO-KHC) wird, getragen durch die teilnehmenden Kliniken, aktive Qualitätssicherung im Sinne der Patienten betrieben und gleichzeitig wissenschaftlich begleitet.
- Das dargestellte Register kann daher beispielhaft für andere Bereiche der medizinischen Versorgung betrachtet werden und setzt damit auch international sichtbare Standards.

### Supplementary Information




