# Supplementary material for: Structure and establishment of the German Cochlear Implant Registry
Source: HNO. 2023 May 17;71(12):767–78. [Article in German] doi: 10.1007/s00106-023-01309-7 (PMC10663275; doi:10.1007/s00106-023-01309-7)
Supplement: Supplementary file 1 [file 106_2023_1309_MOESM1_ESM.pdf]

Datenblöcke CI-Register

1-3: Einmalig angelegte Datenblöcke

**1. Basisdaten**
ID (Code) Versorgende Einrichtung (Klinik):
Patienten-ID Pseudonym:
Geburtsdatum: TT.MM.JJJJ
Sterbedatum: TT.MM.JJJJ
Geschlecht: W/M/D/ka
Muttersprache Deutsch: ja/nein/nicht erhoben

**2.Präoperative Audiometrie (für jede Seite anzulegen)**
Seite: R/L
Tonaudiogramm LL/KL (125/250/500/750/1000/2000/4000/8000Hz): 0-120 dB HL / nicht bestimmbar
Sprachtest:
Freiburger, Hörverlust Zahlen (KH ohne HG, 50%, 100 dB SPL wenn unter 50%): 0-100 dB SPL / nicht erhoben
Freiburger Einsilber (KH ohne HG 65 dB SPL): 0-100 % / nicht erhoben
Freiburger Einsilber mEV (KH ohne HG, mind. 95 dB SPL): 0-100 % / nicht erhoben
Freiburger Einsilber (FF, mit HG 65 dB SPL): 0-100 % / nicht erhoben
Mainzer Kindersprachtest (KH, ohne HG, I/II/III, 65 dB SPL): 0-100% / nicht erhoben
Mainzer Kindersprachtest (FF, mit HG, I/II/III, 65 dB SPL): 0-100% / nicht erhoben
Göttinger Sprachverständnistest (KH, ohne HG, I/II, 65 dB SPL): 0-100% /nicht erhoben
Göttinger Sprachverständnistest (FF, mit HG, I/II, 65 dB SPL): 0-100% /nicht erhoben
Reimtest OlKi in Ruhe (KH, ohne HG, 65 dB SPL): 0-100% / nicht erhoben
Reimtest OlKi in Ruhe (FF, mit HG, 65 dB SPL): 0-100% / nicht erhoben
Satztests (FF, S0N0) mit HG, Sprachpegel 65 dB SPL (Einfachauswahl)
OlSa in Ruhe: 0-100% / nicht erhoben
OlSa Schwelle im Störgeräusch (SRT50): -30 bis +30 dB SNR /nicht erhoben
GöSa in Ruhe: 0-100% / nicht erhoben
GöSa Schwelle im Störgeräusch (SRT50): -30 bis +30 dB SNR /nicht erhoben
HSM in Ruhe: 0-100%/nicht erhoben
HSM Störgeräusch: 0-100 %/nicht erhoben
HSM SNR: -5, 0, +5, +10 dB/nicht erhoben
OlKiSa in Ruhe: 0-100% / nicht erhoben
OlKiSa im Störgeräusch (SRT50): -30 bis +30 dB /nicht erhoben
Objektive Messungen
OAE (TEOAE/DPOAE) nachweisbar: ja / nein / nicht erhoben
BERA nicht freq.-spezifisch (LL/KL): keine Reizantwort / Schwelle in dB nHL / nicht erhoben
BERA/ASSR frequenz-spezifisch (500/4000Hz): keine Reizantwort / 0-120 dB HL / nicht erhoben

3.Präoperative Höranamnese (für jede Seite anzulegen)
Seite: R/L
Zeitpunkt des Hörverlusts: prälingual / perilingual / postlingual / nicht erhoben
Hörverlust (CI-Ohr) in Jahren: 0-1, 1-5, 5-10, 10-20, >20, nicht erhoben
Taubheit (CI-Ohr) in Jahren: 0-1, 1-5, 5-10, 10-20, >20, nicht erhoben
Aktuell Hörgerätenutzung im zu versorgenden Ohr: ja / nein
Versorgung Gegenohr: Normalhörigkeit, Hörminderung (keine Vers., HG, KL-HG, IHG, CI, Sonstige, nicht erhoben)
Ursache der Hörstörung: bekannt: ja / nein / nicht erhoben (wenn ja, Mehrfachauswahl möglich): genetisch, altersbedingt, syndromal, traumatisch, endokrinologisch, toxisch, infektiös, atypische Anatomie Innenohr, atypische Anatomie Hörnerv, atypische Anatomie zentrale Hörbahn, M. Meniere, Vestibularisneurinom, sonstige)
Art der Hörstörung: cochleär / neural / sonstige / nicht erhoben

4-10: Mehrmalig angelegte Datenblöcke

**4.Implantat (für jede Seite/jeden Eingriff anzulegen)**
Seite: R/L
Implantationsdatum: TT.MM.JJJJ
Implantathersteller (1: Advanced B., 2: Cochlear, 3: MedEl, 4: Oticon, 5: Andere): 1-5
Implantat-Bezeichnung: <Liste, Sonstige>
Implantatseriennummer:
Explantation: j/n
Explantationsdatum: TT.MM.JJJJ
Explantationsgrund nach Klassifikation: (B1, B2, C, D,)

**5. Operation (für jede Seite anzulegen)**
Seite: R/L
Operationsdatum: TT.MM.JJJJ
Operationsgrund: 1: Primär-Operation / 2: Revisions-Operation: 1-2
Elektrodeninsertion (1: Rundfenster / 2: Cochleostomie / 3: sonstige): 1-3
Insertionstiefe (1: partiell / 2: vollständig): 1-2
- Intra-OP Funktionskontrollen (Elektrodenimpedanz und/oder E-CAP): regelhaft / nicht regelhaft / nicht erfolgt
Durchführung radiologische Lagekontrolle Elektrode: ja / nein
wenn ja (1: regelrecht / 2: nicht regelrecht / 3: kA); und wenn ja welche Methode<br>(1: konventionelles Rö/2: DVT/3: CT/4: sonstige): 1-4
Revisions-Operation: J/N
Indikation Revisions-Operation: medizinisch, technisch, sonstige, k.A.
Klassifikation Implantatfunktion: A-D
Implantat-Status bei Revisions-Operation 1: Gerät belassen / 2: Wechsel / 3: Explantation: 1-3
Falls Reimplantation: 1,2…n-ter Implantatwechsel

**6. CI-bezogene Komplikationen**
Seite: R/L
Revisionsbedürftige Fehllage der Elektrode: j/n
Fazialisparese: House-Brackmann Grad I-VI
Stationäre Aufnahme war aufgrund CI-bezogener Komplikationen erforderlich: j/n
Meningitis nach CI-Versorgung: j/n
Tod in Verbindung mit der CI-Versorgung: j/n

7. CI-Nutzung und Rehabilitationsfortschritt
Seite: R/L
Implantatfunktion (Klassifikation nach Consensus 2005): A, B1, B2, C, E, nicht erhoben
CI-Nutzungsdauer (Zeit in Std/Tag): 0-24, kA
Nutzungsdauer erhoben durch (1: Patienten-/Elternangabe /2: data logging/ 3: kA): 1-3
Aktueller Rehabilitationsstatus:<br>Prozessphase (1: Basistherapie /2: Folgetherapie / 3: Nachsorge / 4: unbekannt / 5: nicht erhoben): 1-5.

8. Postoperative Audiometrie
Seite: R/L
Zeit nach CI-Operation in Monaten: 0-XX (Automatisch)
Tonaudiogramm (unversorgt) LL/KL (125/250/500/750/1000/2000/4000/8000Hz): 0-120 dB / nicht bestimmbar
Sprachtest (mit CI, FF, Sprachpegel 65 dB SPL):
Freiburger Zahlen: 0-100 %
Freiburger Einsilber: 0-100 %
Mainzer Kindersprachtest (I/II/III): 0-100% / nicht erhoben
Göttinger Sprachverständnistest (I/II): 0-100% /nicht erhoben
Reimtest OlKi: 0-100% / nicht erhoben
Satztests (FF, S0N0) mit CI, Sprachpegel 65 dB SPL (Einfachauswahl)
OlSa in Ruhe: 0-100% / nicht erhoben
OlSa Schwelle im Störgeräusch (SRT50): -30 bis +30 dB SNR / nicht erhoben
GöSa in Ruhe: 0-100% / nicht erhoben
GöSa Schwelle im Störgeräusch (SRT50): -30 bis +30 dB SNR / nicht erhoben
HSM in Ruhe: 0-100%/nicht erhoben
HSM Störgeräusch: 0-100 %/nicht erhoben
HSM SNR: -5, 0, +5, +10 dB/nicht erhoben
OlKiSa in Ruhe: 0-100% / nicht erhoben
OlKiSa im Störgeräusch (SRT50): -30 bis +30 dB / nicht erhoben

9. Hör- /Sprachentwicklung (Kinder)
Verwendung alternativer Kommunikationsmethoden (z.B. DGS, GUK, Talker): ja / nein / nicht erhoben
Auditive Wahrnehmungsentwicklung (z.B. Elternfragebogen): unauffällig / auffällig / nicht erhoben
Kommunikative Entwicklung (z.B. Elternfragebogen): unauffällig / auffällig / nicht erhoben
Fortschritt der Hör- /Sprachentwicklung: regelhaft / nicht regelhaft / nicht erhoben
Status Sprachverständnis: unauffällig / auffällig / nicht erhoben
Status Phonetik-Phonologie: unauffällig / auffällig / nicht erhoben
Status Lexikon-Semantik: unauffällig / auffällig / nicht erhoben
Status Syntax-Morphologie: unauffällig / auffällig / nicht erhoben
Status Kommunikation-Pragmatik: unauffällig / auffällig / nicht erhoben
Sinnesspezifische Förderung / vorbeugende Maßnahme erfolgt derzeit: ja / nein / unbekannt/ nicht erhoben
Pädagogische Einrichtung/Schule: Regel-Kindergarten / Regelschule / andere Einrichtungen / nicht erhoben

10. Lebensqualität
Fragebogen zur Lebensqualität erhoben: NCIQ/ andere / nicht erhoben
Elementare Schallwahrnehmung NCIQ1 (0-100)
Sprach- und Musikwahrnehmung NCIQ2 (0-100)
Kontrolle der eigenen Stimme NCIQ3 (0-100)
Psychosoziale Folgen NCIQ4 (0-100)
Aktivitätsverhalten NCIQ5 (0-100)
Soziale Kontakte NCIQ6 (0-100)
NIIQ Gesamtscore NCIQTotal (0-100)
